# Analysis of the coexistence of gaming and viewing activities in Twitch users and their relationship with pathological gaming: a multilayer perceptron approach

**DOI:** 10.1038/s41598-022-11985-0

**Published:** 2022-05-12

**Authors:** L. Javier Cabeza-Ramírez, Francisco José Rey-Carmona, Mª del Carmen Cano-Vicente, Miguel Ángel Solano-Sánchez

**Affiliations:** 1 Department of Statistics, Econometrics, Operations Research, Business and Applied Economics, Faculty of Law, Business and Economics Sciences, University of Córdoba, Puerta Nueva s/n, 14071 Córdoba, Spain; 2 Department of Applied Economics, Faculty of Social Sciences (Melilla Campus), University of Granada, Calle Santander, 1, 52005 Melilla, Spain

**Keywords:** Health care, Risk factors

## Abstract

The enormous expansion of the video game sector, driven by the emergence of live video game streaming platforms and the professionalisation of this hobby through e-sports, has spurred interest in research on the relationships with potential adverse effects derived from cumulative use. This study explores the co-occurrence of the consumption and viewing of video games, based on an analysis of the motivations for using these services, the perceived positive uses, and the gamer profile. To that end, a multilayer perceptron artificial neural network is developed and tested on a sample of 970 video game users. The results show that the variables with a significant influence on pathological gaming are the motivation of a sense of belonging to the different platforms, as well as the positive uses relating to making friends and the possibility of making this hobby a profession. Furthermore, the individual effects of each of the variables have been estimated. The results indicate that the social component linked to the positive perception of making new friends and the self-perceived level as a gamer have been identified as possible predictors, when it comes to a clinical assessment of the adverse effects. Conversely, the variables age and following specific streamers are found to play a role in reducing potential negative effects.

## Introduction

Over time, video games have become one of the most influential, commercially viable and appealing entertainment media available to society^1,2^. The video game has overcome earlier stereotypes^3^; it is no longer just a simple form of amusement designed especially for children, young people or teenagers^4–6^. Indeed, there is no end to the growth in the applications and uses of video games^7–9^. They have become part of the everyday life of millions of users^10^. Forecasts suggest that by 2023 they will play some kind of role in the lives of two-thirds of the world's population^10,11^. On top of all this, there is the recent emergence of new phenomena that have revolutionised how people enjoy video games: the rise of the professionalisation of this hobby through e-sports^12–15^, and watching video games on live streaming platforms^16–18^. These activities are interrelated on platforms such as Twitch^19^, where users have a multitude of ways to enjoy and share their hobby, whether actively—by streaming their own experiences^20,21^—or passively^19^, for example, by watching others play to observe their skills, following competitions and events, sharing experiences in chats, and learning the ins and outs of old games or new releases^16,22,23^. This aspect of the activity, which is far more social, is accessible to almost anyone with an internet connection^19^.

However, there is a less convivial side associated with video games^24–28^, which is also found on streaming platforms^1,11,29–31^: pathological use that could be linked to the abuse of video games, the internet and live streaming. Among others, it is worth mentioning aspects related to excessive gaming^32,33^, bad online behaviour, harassment or dissemination of inappropriate content^34–36^. Since the 1990s, there has been a growing and extremely complex debate centred on the underlying notion of groups of people who lose control of their behaviour and experience pathological symptoms that affect their daily lives^37^. Recently, a prominent core of the research has focused on the effects of co-occurrence of different activities. Studies such as those by Reer et al.^37^, Sussman, et al.^38^ and Andreassen et al.^39^ reveal the feedback between and co-occurrence of adverse forms of behaviour stemming jointly from the internet, social networks and video games. Nevertheless, despite the growing literature on live streaming services^19,40^, no attempt has thus far been made to identify the elements that are most likely to trigger potential pathological use stemming from the consumption of video games through viewing.

The present study seeks to fill this gap and explore the antecedents of pathological gaming stemming from activities related to live streaming platforms; that is, it examines disruptive use derived from the co-occurrence of video game consumption and viewing. Specifically, the focus is on the motivations for using services of this type, positive perceptions of video game consumption, and characteristics linked to the user's profile as a gamer and viewer (age, self-perceived level as a gamer and viewing time). Furthermore, we analyse inappropriate behaviour linked to making hurtful comments to other users^41,42^. To this end, we review the theoretical background related to the aforementioned independent variables, and develop a multilayer perceptron (MLP) artificial neural network (ANN)^43^. The proposed network is generated from a sample of 970 video game users in Spain, just before the Covid-19 lockdown.

In terms of scope, this paper contributes to the existing literature on the controversial balance between the positive side of video game use and the concern for a counterpoint of pathological components. In this context, this paper stands as one of the first studies to explore excessive use derived from the overlapping of gaming and viewing activities, since previous research has mainly focused on analysing positive or negative uses without considering the co-occurrence of these two closely related activities. From a methodological perspective, the use of artificial neural networks to establish usage patterns contributes to provide a more holistic comprehension, and opens new perspectives to understand the importance of the variables used and to consider future causality analyses, for example, based on structural equations modelling, or experimental design^44–46^. Among the most salient results, it was found that positive perceptions related to the social component of video games have an impact on three of the indicators used by Andreassen et al.^39^ to measure addiction or pathological use: playing or watching video games to forget about problems, feeling bad if you cannot play or watch video games, and spending more and more time playing or watching video games. At the same time, the inverse effect of age on the dependent variables used in the proposed neural network was also highlighted, indicating that, as age increases, most of the indicators of pathological use would be reduced. This would be particularly significant in the context of the existing debate on gaming as a pathology^47,48^, as it suggests that some effects may be circumstantial and tend to diminish as cognitive control over behaviour increases^49^.

## Literature review

### Background

Streaming platforms, with Twitch leading the field, constitute a novel hybrid of a communication media and a vast social network, based primarily around video games^40,50,51^. This type of service offers its users a space to watch content or to live stream, and millions of people connect every day to watch others play or stream themselves^52^. The ever-growing audience has access to search engines to select channels according to their preferences, typically seeking out a particular streamer or a specific video game^51,53^. This new way of experiencing video games encompasses most of the benefits attributed to them, but it also brings with it some of the possible adverse effects related to online video games^1,11,29–31^. These factors can often trigger functional impairment, loss of productivity, poor academic performance, mood disorders and anxiety^54,55^.

When video game enthusiasts start using live streaming platforms, they enjoy new possibilities to improve their experience with video games, better their skills and abilities by watching others play, expand their network of friends, and even professionalise their hobby^56–58^. However, all this requires a significant increase in the time spent on their hobby, now divided between viewing and actually gaming^11^.

### Discussion on the problems associated with video games

Numerous positive effects linked to the use of video games have been described in the field of video games research^59^. Among the most prominent are those related to learning^60^, social support^61,62^, improved attention span, cognitive enhancement and creativity^63–65^. Regarding live streaming, benefits leading to educational, economic, employment or inclusion opportunities have also been found^56,66,67^. Some recent examples can be observed in studies such as those by Formosa et al.^68^ and de Wit et al.^69^ which show how playing and watching video games are configured as a hobby with the capacity to help manage difficult moments in life. However, despite the many positive applications of the range of activities focused on video games, there are concerns, the most important being those associated with a minority of users who may experience transient or persistent symptoms traditionally associated with pathological gaming, for example, changes in mood, tolerance or salience^70^. In this regard, Chen and Chang^31^ note how the inclusion of online gaming addiction in the fifth edition of the Diagnostic and Statistical Manual of Mental Disorders as a tentative disorder (DSM); and the admission by the World Health Organization^71^ of gaming disorder in the International Classification of Diseases were a flashpoint for debate about possible adverse effects. In fact, some authors have expressed concern about the possible stigmatisation of millions of people who use gaming as a healthy hobby, leading to a critical current that warns about the medical, scientific, public health, and social consequences of formalising a disorder in a perhaps premature manner^47,48^.

Ferguson and Colwell^72^ in a recent survey to 214 academics reported that approximately 31% of the researchers participating in their study were sceptical about pathological gaming as a mental health problem. Furthermore, they stated that almost half of them did not believe in the effectiveness of the established criteria for diagnosis (50.3% DSM; 43.5% WHO). An additional problem involves the terminology employed in the empirical research conducted, as there is no general agreement when referring to possible adverse effects, with different terms used to describe very similar concepts (addictive disorder, pathological, problematic, excessive or compulsive use)^73^. A reasonable criticism of current diagnostic approaches lies in the fact that high commitment to an activity, repetitive and entailing prioritisation of activities, is neither problematic in itself, nor necessarily linked to adverse consequences^74^, such as a person who is very committed to sport, cinema or any other hobby in which he or she is particularly involved. In any case, this work aligns with the reasoning of Ferguson (2020) who identifies four points of convergence between the two positions: some individuals may experience gaming excesses; video game problems are infrequent; video game disorders are often accompanied by other types of disorders (e.g., personality disorders); and finally, research on adverse effects is valuable, as it is necessary to further knowledge about the interrelationships between the main variables involved in understanding a phenomenon as multifaceted as video games.

### Pathological use of online video games

The concept of problematic use of the internet, social media and video games is extremely complex^75^. Different meanings are attached to similar phenomena^37,70^, all of which are linked to negative effects that persist for at least a year, compulsive or unhealthy use of digital media^76–78^, and disordered^79,80^, addictive^39,81^, or excessive^30,82^ use. It should be noted that, unlike internet gaming disorder (IGD), problematic social media use (PSU) has not yet been recognised as a disorder by the WHO or in the APA manual^83^. Streaming platforms such as Twitch combine the features of a vast social network with live video game content^19^. Thus, according to Reer et al.^37^, PSU would include outcomes similar to those defined in section III of the fifth Diagnostic and Statistical Manual of Mental Disorders (DSM-5) for IGD: preoccupation and excessive connection; time spent gaming at the expense of other activities such as studying, work, relationships and health; and symptoms such as addiction, loss of control and continued use despite experiencing negative consequences^84^. The concept of problematic use or viewing refers to the user's inability to control the hours spent on (non-work-related) online activities, signs of withdrawal, use to escape social or family problems, and conflicts with the surrounding environment when abandoning other activities^29,31^. An additional problem relates to bad behaviour or aggressiveness on the platform, with hurtful comments or other types of behaviour intended to disrupt the online atmosphere^41,42^.

In line with the aforementioned literature, this study adopts a broad definition of potential problematic use, relating to the various current behaviours that can lead to future problematic use if they persist. These include using or watching video games to forget about real-life problems, experiencing a feeling of distress when not playing, neglecting other important tasks to play or watch, spending more and more time gaming or watching video games^39^, spending more money than expected on video games, and making hurtful comments to other people^11^. Therefore, potential problematic use is understood in a non-clinical sense, but it has the potential to become actual problematic use^85^. In this regard, we will follow the argument of Ferguson, et al.^86^ and we will use the term pathological gaming or use of video games, which has the advantage of being widely used and avoids the terminological concern of making a misleading comparison with substance use.

### The role of the motivation for using live streaming platforms in pathological use

Motivation is one of the aspects that has the strongest influence on the desire to keep playing^87^. Among the most influential motivational factors when gaming or watching others play video games are the perceived utility as a source of escapism and fun, social inclusion and the strengthening of friendship networks, and the need for improvement and information^50,88^. The most widely used theoretical framework for understanding the reasons that lead millions of users to watch others play is the uses and gratification theory^19,40^. This approach provides an explanation for media consumption behaviour^89,90^ based on the motivations for use and rewards that users expect to gain when they use a specific type of communication media. Four key principles underpin the theory: there is an objective and a motivation for using the medium; it is used to satisfy desires or needs; there are social and psychological factors that influence the use of the medium; and lastly, there is a relationship between interpersonal communication and the medium used^91^. According to these assumptions and the live streaming research that has applied this approach^92,93^, there are two main types of gratification related to the use of such platforms. On the one hand, there are the gratifications sought, relating to the instrumental motives for use and expectations about the benefit that will be attained; on the other hand, there are the gratifications obtained, which refer to the needs that are met^90,94^. Both types affect the intensity and frequency of use of such services^94^. Building on this framework, authors have identified as many as five motivations that determine the use of streaming platforms such as Twitch (entertainment, sociability, sense of community, information-seeking and instrumental use)^11,14,50,95^.

Regarding pathological use, the different motivations that lead the user to play are one of the most commonly-used predictors in the assessment of disorders stemming from gaming^96^. In this regard, Khang et al.^33^ note that the user's motives—such as the need to escape, a way of passing the time, entertainment, feeling a sense of community, achievement or satisfaction—are significantly associated with online gaming flow and possible adverse effects derived from intensive use.

In this study, we apply the main motivations identified by Hamari and Sjoblom^14^, Sjoblom and Hamari^50^ and Hilvert-Bruce et al.^95^ to explore the impact of motivation on the potential negative use of live streaming.

### Influence of positive perceptions on pathological use

People can satisfy a wide range of needs by playing or watching video games: for example, needs related to competition, as they attain the necessary knowledge and skills to play the game well; needs related to autonomy, as they feel a sense of ownership of their decisions within the game or when live streaming; and needs related to relationships, affiliation and belonging, as they experience the feeling of being part of a larger community sharing the same interests, or build up their network of friends^97^. This approach is based on the theory of self-determination^98^, which holds that the three basic psychological needs (competence, autonomy and relatedness) provide greater intrinsic motivation and therefore affect the activity to be done. With reference to this framework, Mills et al.^97^ explored whether the perception of meeting these types of needs can become a trigger for undesired effects. Said study Mills et al.^97^ revealed significant and positive correlations between frustration outside the realm of video games and pathological use of video games. Consequently, it could be argued that when people perceive positive uses related to their needs being met by video games, they could seek refuge in video games to compensate for shortcomings in other areas, which in turn could cause them to neglect other tasks to the point of triggering problems associated with excessive gaming activity^11^.

Furthermore, gamers and viewers of video games come to live streaming platforms with certain expectancies: of developing skills and abilities, improving their relationships, learning, and even finding job opportunities and inclusion^15,56–58,66,69,99,100^. The notion of expectancy is grounded in the assumption that behaviour is influenced by personal aspirations and the reinforcing or punitive effects resulting from the behaviour. The origins of this approach lie in social learning and personality theories^101^. Wickwire et al.^102^ drew on this framework to explain inappropriate gambling behaviour in adolescents. In this regard, the use of streaming platforms is reinforced by the user's expectancies (social relations^95^, learning^66^ even the possibility of starting a business^67^). In the long term, this could pose a problem if expectancies related to video games are prioritised over others involved in vital development^103^.

In our analysis, we incorporate the approach of Mills et al.^97^ with that of Wickwire et al.^102^, defining the perception of positive use as: “the anticipation experienced by the user and expectancies linked to the possible perceived benefits of playing video games relating to three specific facets: making friends or strengthening friendships; improving skills or competencies; and the possibility of professionalizing the hobby, which in turn will lead to greater autonomy”^11^.

### Gamer profile and its influence on pathological use

There are different types of video game players or viewers, which have evolved over time. In the 1990s, Bartle^104^ established the following four characteristic profiles: “achievers”, or players focused on tackling challenges; “socialisers”, focused on developing relationships, creating contacts and interacting; “explorers”, motivated by a desire to understand the details of the game and the game world; and “killers”, players predominantly driven by competitiveness. These days, the rise of e-sports^12–15^ and live streaming^16–18^ has introduced a new way to further classify gamers into e-sport gamers and recreational gamers^96^. E-sports gamers considers video games as serious leisure, an activity that lies somewhere between a hobby and work, and are driven by intrinsic, personal and social motivations that entail the gratification of competitive and hedonic needs^96^. These gamers show very high levels of affiliation (need for support and interaction with others), commitment and diversion (need for tension and exciting new experiences)^12,96^. Conversely, recreational gamers engage in their hobby in a calmer, less intense way. In this regard, in the field of esports players, Hedlund^105^ developed a typology based on six psychographic (socialization, positive affect, competition, fantasy/escape, coping, pass/waste time) and behavioural factors, identifying up to five types of players: (1) Competitive, (2) Casual, (3) Casual-Social, (4) Casual-Fun, and (5) Casual-Competitive. Similarly, Cabeza-Ramirez et al.^1^ categorised gamers and viewers according to their personality, playing and viewing time, skill and preferred genres, and identified four profiles: casual, social, hobby, and problematic or pathological. Nonetheless, when considering personal and time-linked characteristics, recent typologies capture the individual's commitment to their hobby, so a high time spent, or a high level of commitment to the hobby should not necessarily be associated with adverse effects^47,74^.

#### Self-perceived level as a gamer

The recent study by Banyai et al.^96^ showed that disruptive behaviour and psychiatric distress can appear in any type of gaming, whether intensive, recreational or professional^96,106^. Similarly, in their analysis of the dynamics of progression in online games, Wu et al.^107^ showed how enhanced skills and experience can cause participants to seek out new challenges in order to experience flow and other benefits. There can be a troubling downside to this, in that participants may develop pathological tendencies^107^. Specifically, their analysis revealed that highly specialised gamers showed significantly higher addiction rates. Moreover, it has recently been shown that recreational gamers who consider themselves experts or quasi-professionals display a worrying spectrum of pathological use and bad behaviour online^1^.

#### Viewing time

Playing and watching video games are two closely linked activities^11^. Applicable under this assumption are self-determination theory^98^, which holds that the satisfaction of the three basic psychological needs (competence, autonomy, relatedness) can explain the time spent watching live streaming, uses and gratifications theory^19,40^ and the framework of expectancies and social learning^101^, which provide an explanation based on the anticipated possibilities and gratifications obtained. In this vein, the matter of time spent playing video games is controversial and it has not been systematically demonstrated that intensive use can have negative connotations^108^. However, in different studies, relationships or effects have emerged that could link the variable with pathologies derived from the use of video games^29–31^, especially as time spent increases and time available for other activities decreases^103^.

#### User's age and gender

**Figure 1:**
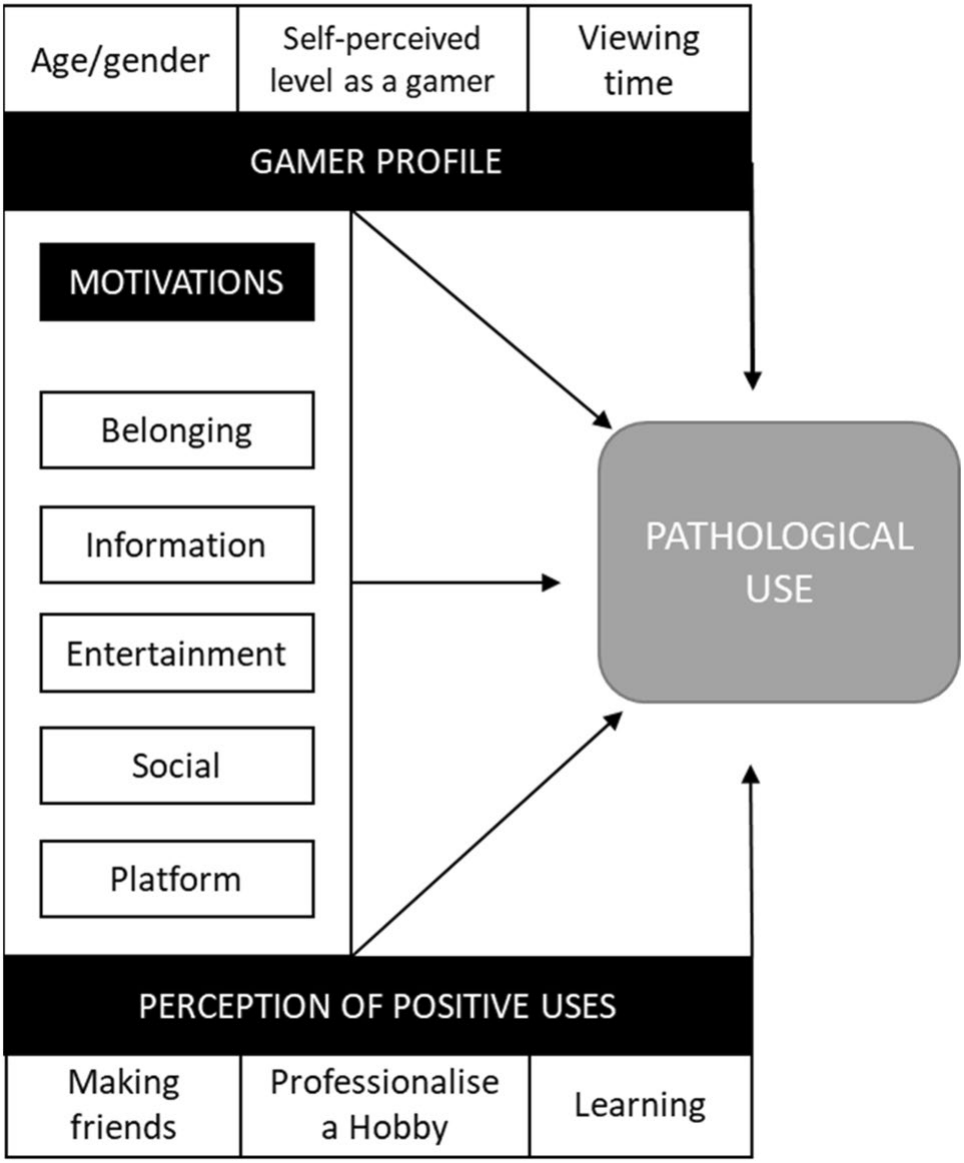
Summary of neural network variables.

Adolescence is considered a period of heightened vulnerability to experience possible overuse of video games due to a lack of maturation in some of the social and emotional regions of the brain, less cognitive control over behaviour and a propensity to seek out emotions^49^. As pointed out by Buiza-Aguado^49^, those adolescents who have less of a perception of the negative consequences of gaming may make decisions that put their psychosocial health—and even their physical health—at risk, due to neglecting important activities such as hygiene, sleep, or eating in order to play video games. There are other important variables related to the user's sociodemographic profile that have long been observed that could be associated with pathological use; for example, the gender of the user, the type of video game^32^, or the time spent^11^. It is commonly believed that men are more intensely involved in multiplayer role-playing games, and tend to be more likely to show problems with video games^109^. However, society and trends are changing at a dizzying pace: there are more and more active gamers, and more cases pointing to a rise in the number of gamers treated for problems with video games^110^, for whom the amount of time spent or types of video game played vary widely^96^. Based on the above, we include both variables and propose the network summarised in Fig. 1.

## Research methodology

### Introduction to the multilayer perceptron approach

**Figure 2:**
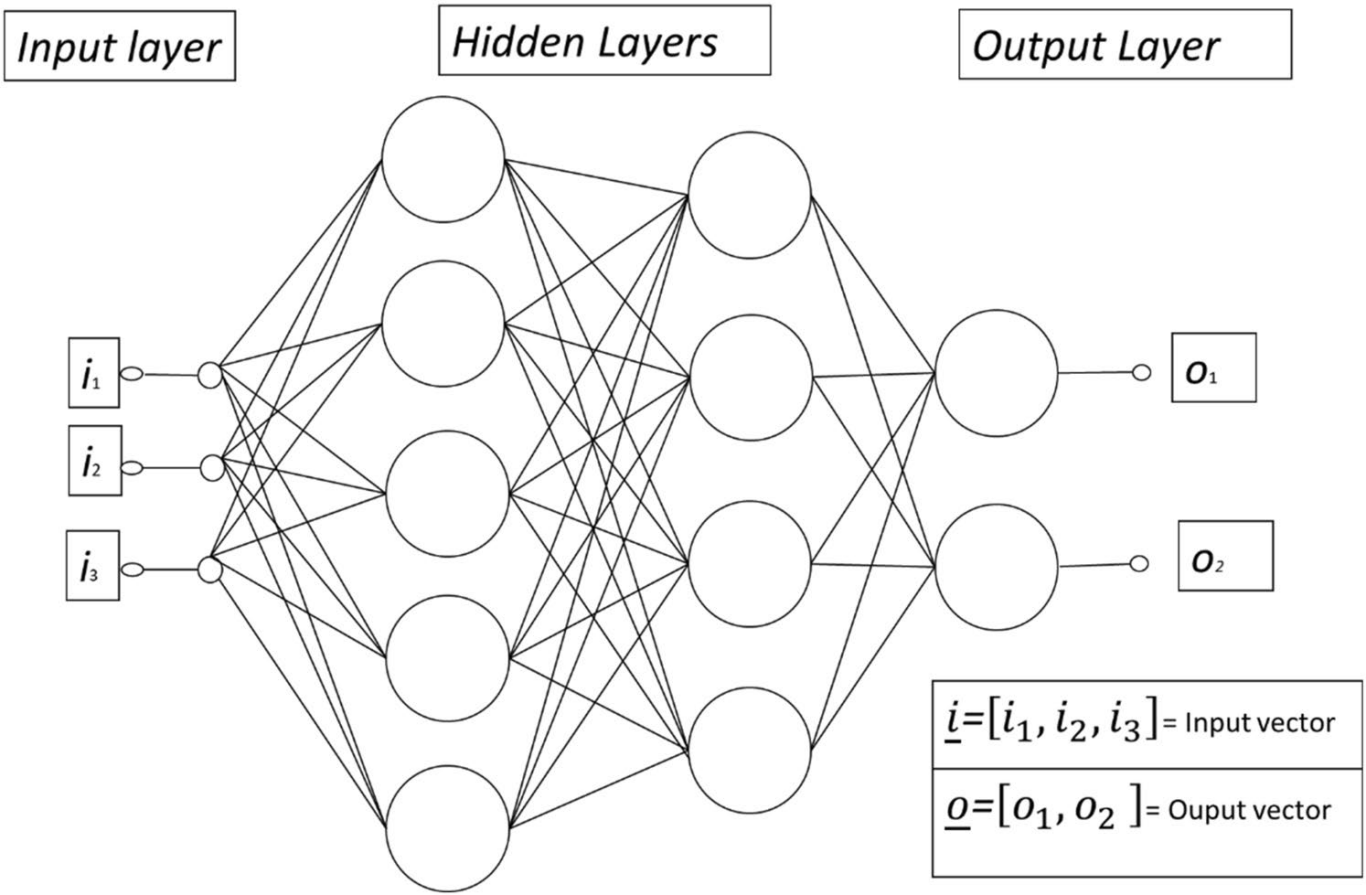
Multilayer perceptron with two hidden layers by Gardner and Dorling^115^.

From a methodological point of view, an ANN is a branch of machine learning (ML), which is an alternative to conventional regression models and other statistical modelling techniques^111^. The nature of ANNs lies in the development of an information processing model similar to the biological system of the human brain. The most remarkable feature of this artificial brain is the possibility of designing a structure capable of processing information by arranging key elements interconnected in the form of neurons, thus working together to provide an answer to a specific problem^111^. ANNs have become a useful tool for classification, pattern recognition, and prediction in different disciplines^44–46,111–114^. The multilayer perceptron approach is a specific type of ANN that is developed through simple interconnected nodes (neurons) representing a non-linear mapping between input and output vectors. It is shown graphically in Fig. 2. Nodes are connected by synaptic weights and output signals which are a function of the sum of the inputs to the node modified by a simple non-linear transfer or activation function. The superposition of multiple transfer functions allows the multilayer perceptron to approximate extremely non-linear functions. The input layer is formed by the network variables (neurons). The hidden layers are composed of the neurons that come from the previous layer and they use as propagation rule the weighted sum of the inputs with the synaptic weights. A transfer function is applied on that sum, which is bounded in response. Finally, the output layer is composed of the neurons whose values correspond to the outputs of the network, showing the results.

Two phases can be distinguished in the implementation of this type of network: the propagation, and the second learning phase. In the propagation phase, the output results of the network are calculated from the input values forwards since this type of network is feed-forward. In the second learning phase, the errors obtained at the output of the perceptron are propagated backwards, with the aim of modifying the weights of the connections, in order to make the estimated values of the network increasingly resemble the real ones. This approximation is accomplished by means of the gradient function of the error. Therefore, ANN has the advantage of detecting both linear and non-linear relationships, and of learning through artificial intelligence functions. The rationale of ANN is to simulate the biological learning behaviour of a brain by means of small computational units, the neurons, arranged in layers connected by digital links, the synapses. The first layer captures external data (for example, theoretically established variables can determine pathological behaviours, or consumer behaviours), and distributes the data to the next layer via links, which are strengthened or weakened following processes defined by mathematical rules. The data is transformed as the neurons propagate the information and the top layer provides a response (for example, possibly problematic consumption patterns). This process, if repeated thousands of times, through feedback mechanisms, allows the neural network to learn the differences between patterns and detect them more accurately.

### Methodological justification

Consequent to the aim proposed, several statistical techniques are considered. For an analysis of the influence of one variable to another, correlation coefficients are commonly used. However, this method only provides information on one isolated variable compared to another. Other methodologies such as hedonic models (regressions) are common in literature, but in studies comparing these models with ANN, the latter tends to present better goodness of fits than linear regressions, due to its nonlinearity, and also a more complex arithmetical procedure than hedonic^44–46^. Additionally, hedonic models have the limitation of only one variable estimation. This reason discards the use of this method as the present research considers various dependent variables (different negative uses) to be influenced by the independent ones selected. For all this, it is considered ANN as a starting point Although the most significant aspects of the proposed methodology are summarised below, further details of its advantages and disadvantages are described in Leong et al.^44^, as well as the main aspects of its application in Leong et al.^45^ and Liébana-Cabanillas et al.^46^.

Rumelhart et al.^43^ define an ANN as a network made up of a series of nodes—or processing elements (PE)—with a certain capacity for storing information. These PE are composed, first of all, ofan input vector (*x*_1_, *x*_2_, …, *x*_*n*_) that includes the independent variables (all of them previously coded to obtain numerical values as a response), with the corresponding synaptic weights (*w*_1_, *w*_2_, …, *w*_n_), which are applied to the input vectors using a propagation rule (based on the corresponding linear combination). An activation function is applied to this result determining the value of these PE, grouped in layers: input layers, intermediate layers (or hidden layers), and output layers that correspond with the estimation of the dependent variables. Neural networks represent a powerful data processing technique that has reached maturity and has been extensively applied^116^. The main appeal of this methodology lies in its remarkable information processing characteristics, nonlinearity, high parallelism, fault and noise tolerance, and learning and generalisation capabilities^112^. ANNs offer a high degree of fit and have previously been applied in different fields in social sciences^117,118^ and health^113,114^, including analyses of types of problematic substance use and health problems^119^. Although the use of ANNs is still new in the field of dependency analysis, their capacity has recently been revealed, especially for the development of hybrid methodological models in combination with structural equations^44^.

The software used to develop the ANN is SPSS Statistics v. 23. The input values correspond to motivations, perceived positive uses, age, gender, self-perceived level as a gamer and viewing time (independent variables), while the output values are potential problematic use (dependent variable). Within ANN, several types of networks coexist, such as the multilayer perceptron (MLP) and the radial basis function (RBF). Several tests are performed taking into consideration both systems, where different topologies and activation functions are tested in the network development process, before finally selecting the ANN that shows the best fit in terms of the coefficient of determination (*R*^2^) and mean absolute percentage error (MAPE), of the MLP type in this case. Further insight into the advantages and disadvantages of ANN implementation can be found in Gardner and Dorling^115^, more details on the methodological application in Basheer and Hajmeer^112^ and Dreyfus^116^, and recent developments to overcome some of their limitations in Leong et al.^45^, Liébana-Cabanillas et al.^46^ and Leong et al.^44^.

### Data collection and sample design

The exploratory descriptive analysis was conducted through convenience sampling, an approach which makes it possible to access relevant information quickly and cheaply^29^. The target population for the study was Spanish people aged over 16 years old. The data were collected via the SurveyMonkey platform during the month of February 2020 (just before the Covid-19 lockdown). A link was generated and distributed on social media, video game forums, and Spanish Twitch channels via text messages encouraging recipients to distribute the questionnaire to their close contacts who were gamers and viewers of video games. The introduction included information on the approximate duration of the survey (10 min), purpose of the research, rights regarding voluntary participation and guarantees of anonymity and confidentiality. It was stated that by clicking to complete the questionnaire, respondents were giving their informed consent. Prior to distribution, the authors consulted the ethics committee of their university (Bioethics and Biosafety Committee and Research Integrity Committee of the University of Cordoba), and the principles and recommendations of the Declaration of Helsinki were observed in the research.

The data collection started with a first draft of the questionnaire, which was reviewed by a group of five researchers to check the logical order of the questions, and the validity and reliability of the measures. A pilot test was then carried out, also through convenience sampling (N = 15), and based on the results, the questions were reformulated. The answers to the pilot test were not included in the final analysis, so as not to bias the results. The final sample consisted of 970 valid responses after excluding questionnaires that did not report viewing time. Apart from this, no further restrictions were imposed on the selection criteria, in order to ensure a high degree of randomness. Table 1 shows a sociodemographic profile consistent with the data provided by the Spanish Videogame Association^120^ (p. 22). The sample is mostly men (approximately 70%) and the most representative age range is 18–25 years old, accounting for almost 50% of respondents. Approximately three out of four respondents work, and most have a mid-level or advanced educational level.

**Table 1:** Respondents’ sociodemographic profile.

Gender (GEN)		Age (AGE)	
Male	69.38%	Under 18	11.24%
Female	30.62%	18–25	48.97%
		26–35	20.21%
		Over 35	19.59%

Educational level		Employment status	
Primary education	3.71%	Full-time work	48.19%
Secondary education	9.07%	Part-time work	29.58%
Upper secondary/VET	55.77%	Work and study	4.55%
University degree	21.75%	Study	13.03%
Postgraduate/Master/PhD	9.69%	Unemployed	4.65%

Table 2 shows the gamer profile for the sample, which again aligns with the data reported by AEVI^120^. It can be seen that time spent gaming exceeds viewing time, and is not particularly high. Gamers who see themselves as having a medium level predominate (33.61%), although 30% consider themselves experts or pro. In order to play and watch, they mainly use smartphones, PCs and PlayStations, and in terms of platforms, Twitch followed by YouTube Gaming. Regarding preferred genres—each respondent was allowed to choose three options—the action and adventure genre stands out. Only 7.53% of respondents reported spending money on streaming platforms, mainly through subscriptions. Lastly, nearly 12% claimed that they live streamed or had done so in the past.

**Table 2:** Respondents' gamer profile.

Weekly hours	Gaming	Viewing (WGV)	Self-perceived level as a gamer (SPL)	
0–3 h	40.00%	69.69%	Novice	26.49%
3–7 h	19.48%	14.23%	Amateur	9.90%
7–10 h	13.71%	6.29%	Regular	33.61%
10–15 h	9.90%	4.95%	Expert	22.78%
15–25 h	9.69%	2.68%	Pro	7.22%
More than 25 h	7.22%	2.16%		

Platforms used for playing (1–5)	Mean	Std. Dev	Favourite genre (3 options max)	
Smartphone	3.12	1.43	Action/adventure	42.99%
PC	3.07	1.66	Real-time strategy	21.65%
PlayStation	2.17	1.45	Adventure	19.18%
Tablet	1.67	1.09	Sport	18.56%
Xbox	1.25	0.76	OnlineFPS	17.84%
Nintendo	1.73	1.20	MMORPG	15.98%
**Streaming** **platforms** **(1–5)**	**Mean**	**Std.** **Dev**	Simulator	15.36%
Twitch	2.24	1.53	Races	15.15%
YouTube Gaming	2.13	1.38	Turn-based strategy	14.74%
Mixer	1.16	0.53	Puzzles	12.68%
Caffeine	1.05	0.33	Music/creative	11.34%
**Do** **you** **spend** **money** **on** **streaming** **platforms?**			MOBA	10.72%
Yes	7.53%		Arcade	10.62%
No	92.47%		JRPG	8.04%
**What** **do** **you** **spend** **money** **on?**			Survival	7.94%
Nothing	88.85%		SingleFPS	6.60%
Subscription	7.64%		**Are** **you** **or** **have** **you** **been** **a** **streamer?**	
Donation	1.75%		Yes	11.98%
Both of the above	1.75%		No	88.02%

### Measurement instrument and scales

The questionnaire included three additional sections. A section dedicated to motivation, in which the measures were adapted from those previously used by Gros et al.^121^, Hilvert-Bruce et al.^95^, and Sjoblom and Hamari^50^. Another section addressing positive perceptions about video games and streaming platforms based on the scales proposed by Wu et al.^122^ and Wickwire et al.^102^, adapted to measure three expectancies: making new friends, supporting education, and the possibility of turning a hobby into a profession. Lastly, pathological use was assessed in a non-clinical sense, adapting the questions from the social media addiction scale provided by Andreassen et al.^39^, and including two specific questions about making hurtful comments^42^ and spending too much money on video games^123^. The variables were measured on a five-point Likert scale. Table 3 summarises the coding, statistics and scales.

**Table 3:** Instrument.

Code	Question	Mean	Std. Dev	Adapted from
**Motivations** **1** **(platform)**				
M101	To follow specific games	2.27	1.43	
M102	To follow specific streamers	2.17	1.42	
M103	To follow tournaments or events	2.07	1.35	
**Motivations** **2** **(entertainment)**				
M201	For entertainment	2.48	1.47	
M202	As an alternative or complement to social networks or TV	2.25	1.39	
**Motivations** **3** **(social)**				
M301	To make new friends	1.32	0.74	
M302	To communicate with other viewers via chat	1.45	0.87	^50,95,121^
M303	To contact a streamer	1.23	0.60	
M304	To watch Twitch with friends	1.44	0.84	
**Motivations** **4** **(information)**				
M401	To learn new gaming strategies	2.27	1.35	
M402	To stay up to date on my favourite video games	2.23	1.38	
**Motivations** **5** **(belonging)**				
M501	I feel like I'm part of the Twitch community	1.48	0.92	
M502	I feel like Twitch is part of today's gaming culture	2.54	1.58	
**Positive** **uses**				
PU01	My video games hobby has helped me make new friends	2.24	1.32	
PU02	Streaming platforms help my education (for example, in languages)	1.98	1.13	^102,122^
PU03	My video games hobby could become my profession	1.55	1.01	
**Negative** **uses**				
NU01	I've played or watched video games to forget about real-life problems	2.30	1.32	
NU02	I've felt bad if I haven't been able to play/watch	1.65	0.97	
NU03	I make hurtful comments to other users	1.40	0.87	^39^
NU04	I've neglected other important tasks (sports, studying, work) to play/watch	1.85	1.07	
NU05	I've spent more money than I expected on video games	1.29	0.54	
NU06	I've been spending more and more time playing/watching video games	2.07	1.14	

## Results: multilayer perceptron development

**Figure 3:**
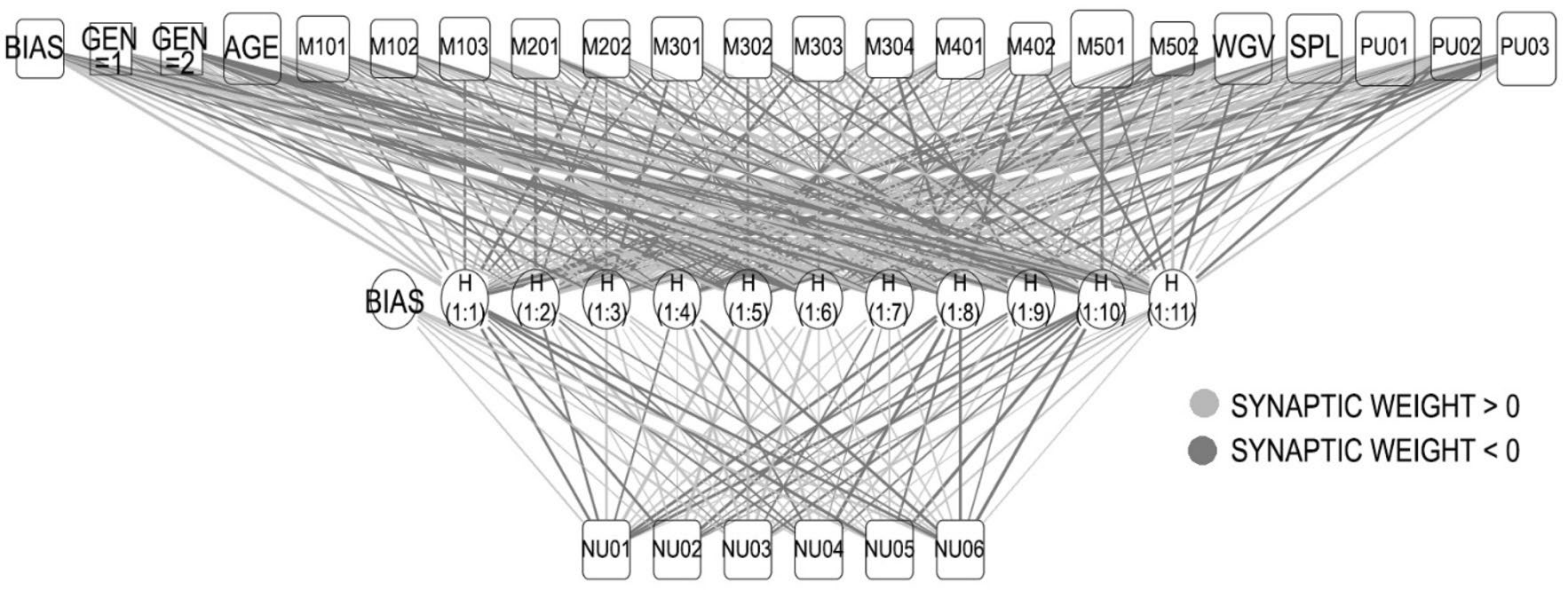
ANN graphic representation.

The architecture of the network is presented in Table 4 and graphically depicted in Fig. 3. Input values, which act as independent variables, were divided into factors (nominal, which are numerically coded) and covariates (numerical). They were all standardised and multiplied by their respective synaptic weights (Fig. 3) in the network. In turn, these values were transformed using a hyperbolic tangent activation function. Finally, these values were multiplied by their corresponding synaptic weights to yield the output values, corresponding to the negative uses, which act as dependent variables. However, since these values were standardised, they must be de-standardised to obtain estimates on a 1–5 Likert scale.

**Table 4:** Network architecture.

Input layer	Factors	1	GEN = 1, male
		2	GEN = 2, female
	Covariates	1	AGE
		2	M101
		3	M102
		4	M103
		5	M201
		6	M202
		7	M301
		8	M302
		9	M303
		10	M304
		11	M401
		12	M402
		13	M501
		14	M502
		15	WGV
		16	SPL
		17	PU01
		18	PU02
		19	PU03
	Number of units (excluding bias)		21
	Rescaling method for covariates		Standardised
Hidden layer	Number of hidden layers		1
	Number of units in hidden layer		11
	Activation function		Hyperbolic tangent
Output layer	Dependent variables	1	NU01
		2	NU02
		3	NU03
		4	NU04
		5	NU05
		6	NU06
	Number of units		6
	Rescaling method for scale dependents		Standardised
	activation function		Identity
	error function		Sum of squares

To produce the ANN, the sample was divided into training and test groups (Table 5). The training group is used to determine the values of the synaptic weights and PE that make up the hidden layer and the output layer. The test group is used to calculate the errors made by the network. When it detects that there is no further decrease in the error, the stopping rule is implemented, and the model is determined. Table 5 shows the relative errors made in this process, as well as the training time and the stopping rule.

**Table 5:** Model summary.

Training (N = 671; 69.2%)	Sum of squares error		1316.504
	Average overall relative error		0.655
	Relative error for scale dependents	NU01	0.622
		NU02	0.663
		NU03	0.781
		NU04	0.663
		NU05	0.649
		NU06	0.552
	Stopping rule used		1 consecutive step with no decrease in error (based on the testing sample)
	Training time		0:00:00.88
Testing (N = 299; 30.8%)	Sum of squares error		603.286
	Average overall relative error		0.724
	Relative error for scale dependents	NU01	0.668
		NU02	0.652
		NU03	0.834
		NU04	0.724
		NU05	0.832
		NU06	0.642

To select the model proposed in the present research, several models were tested, following a better-goodness-of-fit criterion to finally choice one (MLP-7, Table 6). Some of the goodness of fit of the ANN models are shown in Table 6, as more than 20 different model combinations were checked (following both MLP and RBF), and much data about non-convenient models were discarded. Three tests were taken into consideration (Table 6). On the one hand, the mean relative percentage error (MAPE) shows the percentage differences between the absolute values of the actual values and the estimates generated by the network. On the other hand, the coefficient of determination (*R*^2^) indicates the percentage of variability in the actual values that can be explained by the variance in the estimated values. Finally, the root mean square error (RMSE) presents the square root of the variance of the residuals, so the differences between the real and the predicted values (in a 1–5 Likert scale, in this case) in absolute terms. The results (Table 6) are shown broken down by the different dependent variables, the mean values of which are in line with those reported in other social science studies using similar surveys^117,118^, allowing a reliable fit of the model. Additionally, to check MLP selection convenience, RBF’s best model reached in terms of goodness of fit data is included in Table 6. Comparing, lesser MAPE and higher *R*^2^ are obtained in MLP.

**Table 6:** Goodness of fit of the ANN attained.

		NU01	NU02	NU03	NU04	NU05	NU06	Overall
MLP-1	MAPE	46.26%	33.43%	27.92%	35.13%	18.30%	33.16%	32.37%
	*R*^*2*^	42.44%	36.10%	30.60%	39.91%	49.65%	52.38%	41.85%
	RMSE	1.13	0.84	0.85	0.94	0.60	0.93	0.88
MLP-2	MAPE	46.78%	33.95%	31.47%	35.73%	16.88%	34.57%	33.23%
	*R*^*2*^	44.87%	45.25%	31.84%	39.45%	46.47%	46.94%	42.47%
	RMSE	1.13	0.85	0.84	0.94	0.63	0.93	0.89
MLP-3	MAPE	45.42%	32.16%	31.66%	36.20%	18.41%	33.42%	32.88%
	*R*^*2*^	42.29%	38.81%	32.13%	45.00%	49.44%	48.28%	42.66%
	RMSE	1.13	0.85	0.84	0.94	0.61	0.92	0.88
MLP-4	MAPE	47.91%	37.06%	30.00%	38.92%	18.71%	34.72%	34.55%
	*R*^*2*^	44.05%	40.74%	31.50%	47.02%	49.44%	48.27%	43.50%
	RMSE	1.13	0.87	0.85	0.96	0.63	0.94	0.89
MLP-5	MAPE	44.56%	32.66%	29.23%	37.68%	18.05%	33.57%	32.62%
	*R*^*2*^	45.44%	38.31%	31.44%	40.53%	48.18%	49.76%	42.28%
	RMSE	1.10	0.83	0.83	0.94	0.62	0.92	0.87
MLP-6	MAPE	46.41%	31.55%	28.59%	37.49%	19.11%	33.34%	32.75%
	*R*^*2*^	47.99%	37.57%	30.35%	39.99%	48.52%	51.09%	42.59%
	RMSE	1.12	0.84	0.86	0.95	0.63	0.90	0.88
MLP-7	MAPE	44.37%	31.36%	29.30%	35.73%	17.65%	34.02%	32.07%
	*R*^*2*^	41.43%	41.38%	32.18%	42.26%	49.05%	55.37%	43.61%
	RMSE	1.09	0.82	0.83	0.91	0.61	0.90	0.86
RBF-1	MAPE	51.45%	38.20%	30.78%	41.99%	18.27%	39.40%	36.68%
	*R*^*2*^	34.60%	32.71%	31.77%	33.97%	46.71%	44.52%	37.38%
	RMSE	1.17	0.90	0.84	1.00	0.63	0.99	0.92
RBF-2	MAPE	47.41%	32.55%	31.15%	39.12%	21.18%	36.72%	34.69%
	*R*^*2*^	36.95%	31.29%	31.62%	40.08%	51.21%	42.31%	38.91%
	RMSE	1.14	0.88	0.86	0.96	0.62	0.95	0.90
RBF-3	MAPE	50.26%	34.92%	29.30%	38.30%	18.68%	33.35%	34.14%
	*R*^*2*^	39.23%	25.79%	30.84%	35.95%	47.51%	43.01%	37.05%
	RMSE	1.15	0.89	0.86	0.97	0.63	0.96	0.91
RBF-4	MAPE	50.10%	36.23%	26.72%	38.76%	19.43%	35.00%	34.37%
	*R*^*2*^	38.11%	36.59%	29.48%	40.30%	48.19%	50.72%	40.57%
	RMSE	1.15	0.87	0.87	0.96	0.64	0.93	0.90
RBF-5	MAPE	47.32%	34.07%	30.99%	38.60%	19.26%	34.56%	34.13%
	*R*^*2*^	39.27%	35.10%	31.54%	40.08%	49.50%	46.73%	40.37%
	RMSE	1.14	0.84	0.85	0.96	0.62	0.93	0.89

**Figure 4:**
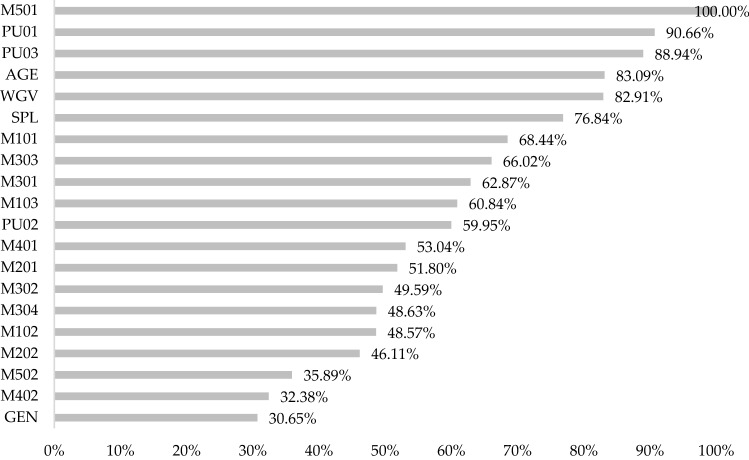
Normalised importance of independent variables in the ANN model.

The ANN attained allows a detailed analysis of the importance of each of the independent variables in the network (Fig. 4). Thus, the feeling of belonging to Twitch (M501), positive uses such as making friends (PU01) or the possibility of transforming a hobby into a profession (PU03), and items related to gamer profile such as age, hours spent watching (WGV) and self-perceived level (SPL) contribute decisively to the model. Conversely, factors such as gender (GEN), the motivation of staying up-to-date on video games (M402) and the perception that Twitch or other streaming platforms are part of the current gaming culture (M502) are among the least influential in the network.

Finally, the model can be used to estimate the individual effects of the independent variables on the dependent variables. This involves taking the estimates of pathological use (NU01 to NU06) when each of the independent variables is at its minimum or maximum value, respectively, while the rest of the input values remain as mean values, or the majority in the case of nominal variables. Table 7 shows the most significant results obtained, presenting the 10 independent variables that had the greatest effect (direct and inverse) on the dependent variables.

**Table 7:** Items with the greatest influence on negative uses.

Direct						Inverse					
↑	PU01	→	↑	NU01	25.57%	↑	AGE	→	↓	NU06	− 21.42%
↑	SPL	→	↑	NU06	22.41%	↑	AGE	→	↓	NU04	− 21.06%
↑	PU02	→	↑	NU03	18.51%	↑	M102	→	↓	NU01	− 17.54%
↑	M501	→	↑	NU03	17.76%	↑	AGE	→	↓	NU02	− 15.57%
↑	PU01	→	↑	NU02	17.62%	↑	AGE	→	↓	NU03	− 14.30%
↑	M101	→	↑	NU06	16.96%	↑	M102	→	↓	NU03	− 13.65%
↑	PU01	→	↑	NU06	15.58%	↑	M102	→	↓	NU02	− 13.21%
↑	SPL	→	↑	NU01	15.46%	↑	AGE	→	↓	NU01	− 13.14%
↑	M502	→	↑	NU01	14.58%	↑	M102	→	↓	NU06	− 12.34%
↑	M103	→	↑	NU03	14.15%	↑	M301	→	↓	NU04	− 12.08%

For example, it can be seen that as the perception that video games have helped the respondent to create new friendships increases (PU01), there is also an increase in negative feelings caused by not being able to play (NU02), the use of video games to escape real life (NU01), or the perception that the respondent’s use of video games is increasing (NU06). These last two effects also increase as the self-perceived level as a gamer (SPL) does. On the other hand, hurtful comments to other users (NU03) increase in line with respondents' perception that the platforms contribute to their education (PU02), the feeling of belonging to the community (M501) and following tournaments and events (M103).

In terms of inverse relationships, it is significant that as the age of the respondent increases, we see a decrease in all the negative uses except NU05. It is also noteworthy that an increase in respondents' interest in following specific streamers (M102) leads to a reduction in several negative uses, such as the use of video games to escape real life (NU01), making hurtful comments (NU03), negative feelings when unable to play (NU02) and the perception of the growing use of video games (NU06).

## Discussion and conclusions

The increasing digitalisation of everyday life offers obvious benefits, but could have a dark side^37^. The exact number of users affected by IGD is currently unknown. The recognition of pathological gaming as a health problem is even questioned^47,124^, and misdiagnosis can occur due to a high commitment to the hobby^74^. One of the largest meta-analyses carried out suggests global prevalence rates of around 3%. Nevertheless, there is significant variability in these estimates due to a higher proportion of analyses being carried out with samples of adolescents, or the use of different measurement scales^125^. Another important aspect concerns the influence of the co-occurrence of linked activities on potentially problematic use. This has been shown by Reer et al.^37^, Sussman et al.^38^, and Andreassen et al.^39^, who demonstrate feedback between and coexistence of adverse forms of behaviour stemming from the combined use of the internet, social networks and video games. Our results add to the emerging research in the field of live streaming and align with recent studies about possible adverse effects^11,29–31^. We can highlight the following findings:

First, we found that the social component linked to the positive perception of making new friends (PU01) is related to three variables that are important when it comes to a clinical assessment of the pathological use^39^, specifically, playing or watching to forget real-life problems (NU01), feeling bad when unable to play or watch (NU02), and spending more and more time playing or watching video games (NU06). This result does not seem surprising given that platforms like Twitch are fundamentally social by nature^126,127^. In this regard, Hilvert-Bruce et al.^95^ note that the amount of time viewers dedicate to live streams is significantly explained by social interaction and a lack of external support. This finding is consistent with those reported by Mills et al.^97^, who showed significant and positive correlations between frustration outside the sphere of video games and pathological use of video games. From a practical standpoint, the results appear to confirm that social support from parents, family and friends could be an extremely useful tool for preventing possible disorders derived from video games^28^.

Second, we found that self-perceived level as a gamer (SPL) is a variable worth accounting for in the assessment of pathological use. Specifically, we observe that it is related to the following variables: spending more and more time watching or playing (NU06) and playing or watching video games to forget about real-life problems (NU01). In this respect, users' gaming skills and the progress they make as they play (at least as they perceive it) are rarely included in analyses of disruptive behaviour. The relationships identified are in line with the theory of social learning and expectancies; that is, as Wickwire et al.^102^ claimed, if the player feels that he/she is making progress when using the live stream, and is continually improving, he/she may prioritise this type of activity to the point where it becomes excessive. In practice, actions could be taken to raise awareness among gamers or viewers, targeted by ability groups; a casual novice gamer is unlikely to have the same expectancies and thus experience the same problems as someone who has a higher level, especially if that gamer feels that he/she is making ever greater progress.

Furthermore, the motivational variables related to being part of a community (M501), following tournaments and events (M103), and even the positive use linked to the influence of video game platforms on respondents' education (in languages, for example—PU02) are related to the probability of making hurtful comments to other users (NU03). In this respect, there have been a fair few studies that have warned of the toxicity of the comments and the adverse environment generated in certain online communities^28,41,42^. Hilvert-Bruce and Neill^41^ claimed that harassment is viewed as normal and is even tolerated in these contexts. Similarly, Lee et al.^128^ reported that verbal attacks are common in multiplayer online games. Their results suggest that there is a certain propensity to make hurtful comments in the competitive sphere—for example, in tournaments and events—when the user feels that he/she is learning to improve and forms part of a larger group involved in a large League of Legends (LoL) tournament. From a practical point of view, this suggests that organisers of events and platforms should introduce measures aimed at raising public awareness to maintain a good gaming environment and prevent any type of aggression, especially in environments of strong commitment to video games.

Fourth, it is striking to note that the variable viewing time (WGV) does not seem to be related to pathological effects; that is, negative potentialities do not emerge as viewing time increases. This may be due to the fact that the variable time spent gaming was not included in the neural network, since the intention was to explore the negative potentialities stemming from viewing. Therefore, future research should explore the cumulative effects of both times (gaming and viewing) on the potential negative effects of the use of video games. As noted by Cabeza-Ramírez et al.^11^, users who perceive positive effects of playing video games might be more likely to develop negative behaviours through the time they spend playing and less through watching, as they prioritise the first activity. This finding thus points to a need for further empirical research on the co-occurrence of watching and playing.

Lastly, the variables following specific streamers (M102) and age—especially the latter—were found to reduce pathological effects. This indicates that instrumental use of the platform does not in itself generate negative potentialities, but it does when accompanied by certain aspects of the user's personality^129^, and when video games fulfil certain expectancies and needs that are not fully satisfied outside of this sphere^97,130^. The case of age was especially notable as it influences a number of variables: spending more and more time playing/watching video games (NU06), neglecting important tasks (NU04), feeling bad when unable to play or watch (NU02), making hurtful comments to other users (NU03) and playing or watching video games to forget real-life problems (NU01). To an extent, this underscores the belief that some pathologies caused by video games in people's lives can be contextual, and they tend to decrease as cognitive control over behaviour increases^49^. In addition, longitudinal research on IGD in studies carried out with samples of adolescents has shown the emergence of transient gaming disorders, characterised by self-recovery^131^.

### Implications

The empirical analyses carried out in this paper add to the emerging research on public health, video games, live streaming and disruptive behaviour^19,40^. The study makes three novel contributions relative to previous studies. First, it includes variables related to the user's gamer profile, self-perception or experience as a player, and viewing time^2,18^. Second, it accounts for the positive expectancies of the video game player-viewer as possible triggers of pathological use^11,97,98^. Finally, the methodology applied, neural networks, is a powerful data processing technique^116^, whose main benefits include nonlinearity, high parallelism, fault and noise tolerance, as well as learning and generalisation capabilities^112^. This type of analysis is starting to be used to optimise decision-making based on cohort studies of substance use disorders^119^. In the near future, it seems likely that ANN studies in the field of live streaming research will have a place in detecting patterns of live streaming consumption and usage. Recent research is adopting a two-step hybrid approach, using structural equation modelling to establish relationships and directions of causality, and neural networks to optimise prediction and pattern detection, as for example Lo et al.^132^ apply this hybrid approach to the analysis of the motives underlying impulse buying during live streaming. However, the choice of approach depends on the complexity and degree of theoretical understanding of the problem. In this case, as has been highlighted throughout the paper, there is an open debate about pathological gaming as a mental health problem. The set of variables that are most significant in assessing the differences between engaged and pathological use are also not precisely known^74^. The multilayer perceptron technique, unlike other statistical techniques, does not make prior assumptions about the distribution of data, can model non-linear functions and can be trained to generalise accurately if new data become available^115^, making it a valid alternative for shedding light on the understanding of the variables involved in such a complex phenomenon^115^.

### Limitations and future research

The present research is not without its limitations, though some of them represent interesting opportunities for future research. First, this is an initial, exploratory approach with a novel methodology (in this field of research); accordingly, it is limited to a set of variables that could be determinants of pathological use. There is thus a need to confirm the results using other approaches such as data mining, regression models and other statistical analyses^111^. Additionally, the proposed neural network can be expanded to include other variables such as time spent gaming, preferred genre, and variables related to the user. In this regard, the cross-sectional approach used limits the scope of the research. Furthermore, the observed relationships between network variables could be caused by the influence of other factors that have not been considered in the research, requiring some caution in interpretation. This implies the need to include in future research additional statistical controls with the inclusion of control variables (for example, educational stress, family support, unemployment, and others), that is, part or all the observed relationships could be explained by other variables. Therefore, future research should consider the use of neural networks combined with structural equation modelling, which would allow determining causal relationships as well as increasing the reliability and validity of the results^44–46^. Second, the concept of pathological use was applied in a non-clinical sense, pointing to future experimental designs in which the effect of the variables included here can be compared between people who have been clinically diagnosed and those who do not suffer any problems stemming from the use of video games. According to Gardner and Dorling^115^, one of the limitations of this methodology lies in the decisions associated with the construction of the network, i.e. the number of layers and nodes. There are no specific rules in this respect, the inputs and outputs are determined by the problem in question. The optimal number of nodes in the hidden layers is related to the complexity of the input–output mapping, the noise of the data and the number of cases available for training. Future research should focus on constructing a network to detect patterns of real or clinical pathological use. This will require feeding the network with a training dataset divided into several sets, including a validation and a test set. The validation dataset should be composed of data from actual diagnosed users. Third, this cross-sectional quantitative research was conducted using a relatively large convenience sample; that is, the findings might not be generalisable, even though the profile of the sample resembles that reported by AEVI^120^. As such, future studies should apply stricter sampling criteria; for example, stratified random sampling. Furthermore, there is still limited research on the convergence between the active use of video games and watching^11^.

Lastly, this is a static study that captures possible variables at a given time, which opens the door to new longitudinal analyses that address the evolution of the user as a player and viewer of video games^49^.

## Data Availability

The datasets used and/or analysed during the current study available from the corresponding author on reasonable request.
